# Assessment and application of non-technical skills in robotic-assisted surgery: a systematic review

**DOI:** 10.1007/s00464-024-10713-1

**Published:** 2024-03-11

**Authors:** Vimaladhithan Mahendran, Laura Turpin, Matthew Boal, Nader K. Francis

**Affiliations:** 1 MSc Patient Safety and Human Clinical Factors, University of Edinburgh, Edinburgh, UK; 2 Department of General Surgery, Yeovil District Hospital, Yeovil, UK; 3 Division of Medicine, BSc Applied Medical Sciences, University College London, London, UK; 4 Division of Surgery & Interventional Science, Royal Free Hospital Campus, University College London, London, UK; 5 The Griffin Institute, Northwick Park Hospital, Northwick Park and St Mark’s Hospital, Y Block, Watford Rd, Harrow, HA1 3UJ UK; 6 Wellcome/EPSRC Centre for Interventional and Surgical Sciences, Charles Bell House, University College London, London, UK

**Keywords:** Human factors, Assessment, Robotic surgery, Surgical training, Non-technical skills

## Abstract

**Background:**

Undeniably, robotic-assisted surgery (RAS) has become very popular in recent decades, but it has introduced challenges to the workflow of the surgical team. Non-technical skills (NTS) have received less emphasis than technical skills in training and assessment. The systematic review aimed to update the evidence on the role of NTS in robotic surgery, specifically focusing on evaluating assessment tools and their utilisation in training and surgical education in robotic surgery.

**Methods:**

A systematic literature search of PubMed, PsycINFO, MEDLINE, and EMBASE was conducted to identify primary articles on NTS in RAS. Messick’s validity framework and the Modified Medical Education Research Study Quality Instrument were utilised to evaluate the quality of the validity evidence of the abstracted articles.

**Results:**

Seventeen studies were eligible for the final analysis. Communication, environmental factors, anticipation and teamwork were key NTS for RAS. Team-related factors such as ambient noise and chatter, inconveniences due to repeated requests during the procedure and constraints due to poor design of the operating room may harm patient safety during RAS. Three novel rater-based scoring systems and one sensor-based method for assessing NTS in RAS were identified. Anticipation by the team to predict and execute the next move before an explicit verbal command improved the surgeon’s situational awareness.

**Conclusion:**

This systematic review highlighted the paucity of reporting on non-technical skills in robotic surgery with only three bespoke objective assessment tools being identified. Communication, environmental factors, anticipation, and teamwork are the key non-technical skills reported in robotic surgery, and further research is required to investigate their benefits to improve patient safety during robotic surgery.

Undeniably robotic-assisted surgery (RAS) has become very popular in recent decades. So far, over 7.2 million RAS procedures have been performed by 2019 since the US Food and Drug Association (FDA) approval in 2000 [1]. In all the commercially available RAS systems today, the surgeon is physically disconnected from the patient and the rest of the surgical team, which is very different from the traditional operating theatre (OR) setup, and this has introduced several challenges to how surgeons and their teams’ function, especially to communication, teamwork and situational awareness. On certain platforms, the robotic surgeon may be working several meters away from the patient, which potentially places considerable limitations on their interactions with OR team. Moreover, robotic system components have a considerable footprint and can restrict movement and obstruct the direct line of sight between team members [2]. The immersive environment of the RAS inadvertently affects the surgeon’s situational awareness, which could negatively impact the surgeon’s decision-making [3] and preclude effective communication between operating staff.

Ever since a report by the Institute of Medicine in 1999 highlighting human errors and their consequences in healthcare, Non-Technical Skills (NTS) have been identified as an essential pillar of patient safety [4]. Studies suggest that up to 60% of surgical patients may be involved in adverse events and breakdown in communication was the cause of 43% of errors during surgery [5]. Flin et al. defined NTS as ‘the cognitive, social and personal resource skills that complement technical skills, and contribute to safe and efficient task performance [6]. While there has been a great emphasis on training, assessment and credentialling of surgeons’ technical competencies in RAS, Non-Technical Skills (NTS) have received less emphasis [7]. As with the introduction of any new medical technology, it is crucial to understand NTS specific to RAS and the state of NTS training for RAS teams. Particular emphasis should focus on preventing errors and response to emergency situations including device malfunction, major haemorrhage or air embolism which may require rapid conversion to open surgery.

In 2019, Kwong et al. reported a systematic review to understand NTS in RAS and how it could be assessed [8]. This review however was limited to robotic urological surgery and highlighted the paucity of tools available for assessing NTS in RAS, and most of them were not specific to robotics. Another older review identified key NTS and their assessment in minimally invasive surgery (MIS) teams but did not include RAS teams [9]. A recently published review by Cha et al. identified objective metrics for measurement in the surgical environment, including RAS, but focusing only on the physiological matrix without assessing NTS [10].

This systematic review aimed to update the evidence on the role of NTS in robotic surgery with a specific focus on evaluating assessment tools and their utilisation in training and surgical education in robotic surgery.

## Methods

A systematic literature review was performed as per the PRISMA (Preferred Reporting Items for Systematic Review and Meta-Analysis) Guidelines [11]. The study has been registered with Research Registry, identification number: review registry 1654.

## Eligibility criteria

The PICOS (Population, Intervention, Comparator, Outcomes and Setting) framework was used to create a well-formulated research question to guide the systematic review (Table 1).

**Table 1:** PICOS (population, intervention, comparator, outcomes and setting) statement

Population	Robotic surgery team- Surgeon, Anaesthetist, Scrub Practitioner, Trainee surgeon
Intervention	1.Key NTS (Non Technical Skills), for example communication, teamwork, leadership, situation awareness and decision making 2. Training or assessment of NTS A. Can be subjective, for example checklist or survey-based tools B. Can be objective, for example Content-coded communication metrics, workflow metrics and physiological metrics
Comparator	None or other non-robotic assessment tools
Outcomes	1.Identify currently available tools for subjective and/or objective assessment of NTS in RAS (Robotic Assisted Surgery) 2. Evaluate the validity and reliability of the assessment tools available and feasibility of use 3. Investigate effect of NTS Training on staff’s knowledge, attitude, behaviour and patient outcomes
Setting	During robotic surgery (intra-operative) or in a simulated setting

Studies written in the English language involving the identification, assessment or training of NTS skills in individuals and teams during live or simulated RAS procedures were included in the review. Study types included cross-sectional, cohort, qualitative studies, non-randomised and randomised control trials.

Minimally invasive surgery other than RAS, robotic surgery without general anaesthesia (as they would not involve the entire team) and studies solely on the evaluation of technical skills, were excluded. Articles without empirical evidence, abstracts without full-text articles, duplicate publications and articles without an English translation were also excluded from the review.

A search of the PubMed, PsychINFO, Medline and Embase databases was conducted in December 2022. Studies up to 1985 were included when a robot was first used in a surgical procedure [12]. Key concepts used in the search were ‘Non-Technical Skills’, ‘Robotic Surgery’, ‘Subjective Assessment’, ‘Objective Assessment’, ‘Robotic Surgery Team’ and ‘Outcome’. Table 2 shows the search terms and strategy used.

**Table 2:** Search strategy and MeSH (medical subject heading search terms)

Question 1: What are the tools that are currently available to assess non-technical skills in robotic surgery?	
Concept 1: Non-Technical Skills	non-technical [tw] OR “non-technical skills” [tw] OR nontechnical [tw] OR “soft skills” [tw] OR “social skills” [tw] OR “human factor*” [tw] OR teamwork [tw] OR “team work” [tw] OR “situation awareness” [tw] OR “situational awareness” [tw] OR vigilance [tw] OR monitor* [tw] OR “cognitive workload” [tw] OR “Team-based learn*” [tw] OR “Team intervention*” [tw] OR “Team train*” [tw] OR “Crew resource management, healthcare” [tw] OR “interdisciplinary communication” [MeSH] OR “interprofessional relations” [MeSH] OR “communication” [MeSH] OR “leadership” [MeSH] OR “decision making” [MeSH] OR “awareness” [MeSH] OR “Metacognition” [MeSH] OR “Cognition” [MeSH] OR “Wakefulness” [MeSH] OR “Cooperative Behavior” [MeSH] OR “Group Processes” [MeSH] OR “Clinical Competence” [MeSH] OR “Mentoring” [MeSH] OR “teach-back communication” [MeSH] OR “ergonomics” [MeSH]
Concept 2: Robotic Surgery	Automation [tw] OR “Robotic Surgical Procedures” [MeSH] OR “Robotic Surgery” [tw] OR “Robotic Assisted Surg*” [tw] OR “Robot Assisted Surg*” [tw] OR “Robotic-Assisted Surg*” [tw] OR “Robot-Assisted Surg*” [tw] OR “Robot Enhanced Surg*” [tw] OR “Robot-Enhanced Surg*” [tw] OR “Surgery, Computer-Assisted”[Mesh]
Concept 3: Subjective assessment	Assessment [tw] OR Tool* [tw] OR Score* [tw] OR Scoring* [tw] OR “Self-Assessment” [tw] OR “Video Recording” [tw] OR “Health Care Evaluation Mechanisms” [MeSH] OR NOTSS [tw] OR “Nontechnical Skills for Surgeons” [tw] OR “Oxford NOTECHS II” [tw] OR OTAS [tw] OR “ICARS” [tw] OR “GEARS” [tw] OR “GERT” [tw] OR “Generic Error Rating Tool” [tw]
Concept 4: Objective assessment	Physiological OR behavior* [tw] OR behaviour* [tw] OR assess [tw] OR evaluation [tw] OR objective [tw] OR measure [tw] OR empirical [tw] OR quantitative [tw] OR “heart rate variability” [tw] OR “HRV” [tw] OR “ECG” [tw] OR “EKG” [tw] OR “skin conductance” [tw] OR “skin conductance level” [tw] OR “SCL” [tw] OR “electrodermal activity” [tw] OR “EDA” [tw] OR “GSR” [tw] OR ocular [tw] OR eye-tracking [tw] OR “eye tracking” [tw] OR “brain measure” [tw] OR “brain activity” [tw] OR “EEG” [tw] OR speech [tw] OR interaction [tw] OR gesture [tw] OR “movement” [tw] OR “heart rate” [MeSH] OR “heart rate determination” [MeSH] OR “electrocardiography” [MeSH] OR “galvanic skin response” [MeSH] OR “blood pressure” [MeSH] OR “blood pressure determination” [MeSH] OR “eye movements” [MeSH] OR “saccades” [MeSH] OR “electroencephalography” [MeSH] OR “feedback, sensory” [MeSH] OR “communication methods, total” [MeSH] OR “manual communication” [MeSH]
Question 2: What is the effect of non-technical skills training on staff’s knowledge, attitude, behaviour and patient outcomes?	
Concept 5: Robotic surgery team	Surgeon* [tw] OR Clinician* [tw] OR anaesthetist* [tw] OR anesthetist* [tw] OR anaesthesiologist* [tw] OR anesthesiologist* [tw] OR “scrub practitioner*” [tw] OR “Scrub nurse*” [tw] OR trainee* [tw] OR Resident* [tw] OR Student* [tw] OR education [tw] OR “Patient Care Team” [MeSH] OR “Simulation Training” [MeSH] OR “authoritarianism” [MeSH] OR “professional practice” [MeSH] OR “delegation, professional” [MeSH] OR “Operating Room Technicians” [MeSH] OR “Operating Rooms” [MeSH]
Concept 6: Outcome	“Practice-Based Learning” [tw] OR Improvement [tw] OR “Patient Safety” [MeSH] OR “Risk Management” [MeSH] OR “Organizational Culture” [MeSH] OR “Task Performance and Analysis” [MeSH] OR “clinical competence” [MeSH] OR “professional competence” [MeSH] OR “professionalism” [MeSH] OR “mental processes” [MeSH] OR “problem solving” [MeSH] OR “Curriculum” [MeSH] OR “Internship and Residency” [MeSH] OR “Medical Errors”[MeSH]

### Screening

Two independent reviewers searched the databases, selected titles, reviewed abstracts and short-listed studies which met the inclusion criteria*.* Any disagreements during study selection were resolved by consensus between the two reviewers.

Full-text review of all the studies which meet met the inclusion criteria were reviewed independently by both reviewers and data extracted. The following data fields were extracted:

1. *Study characteristics—*Authors, year, single or multi-centre, registration/ID, country, name of article, study design, meets the inclusion criteria (yes/no), study setting (dry simulation lab, wet simulation lab, simulated OR, intra-operative), the total number of participants, participant level of experience (Novice, Intermediate, Expert, Unspecified), study funding sources and possible conflicts of interest of the authors.
2. *Evaluation and outcome characteristics—*Name of the assessment tool, type (subjective or objective), NTS domain or construct tested, evaluator type (Self-rated, Novice, Expert, Crowd-sourced, Not applicable/ other), the content of the intervention, duration, intensity and timing, effects of NTS training/ assessment on staff’s knowledge, attitude, behaviour and patient outcomes.

## Data analysis and quality of literature and validity evidence

1. Selected articles were judged on their level of evidence using the OCEBM (Modified Oxford Centre for Evidence-Based Medicine) Working Group Level of Evidence [13].
2. MMERSQI (Modified Medical Education Research Study Quality Instrument) was used to appraise the methodological quality of the studies. Studies have scored a minimum of 23.5 and a maximum of 100 based on 12 outcomes based on the domains of design, sampling, setting, type of data, the validity of assessment, data analysis and outcomes [14].
3. Validity of the judgements made by different NTS assessment tools was evaluated using Messick’s validity framework [15]. Validity evidence was categorised into content, response process, internal consistency, relationship to other variables and consequences [15].

Data management: Covidence systematic review software, Veritas Health Innovation, Melbourne, Australia, a. Available at www.covidence.org was used for deduplication, screening, full-text review, and data extraction. The data synthesised here was then exported to Excel files.

## Results

**Fig. 1:**
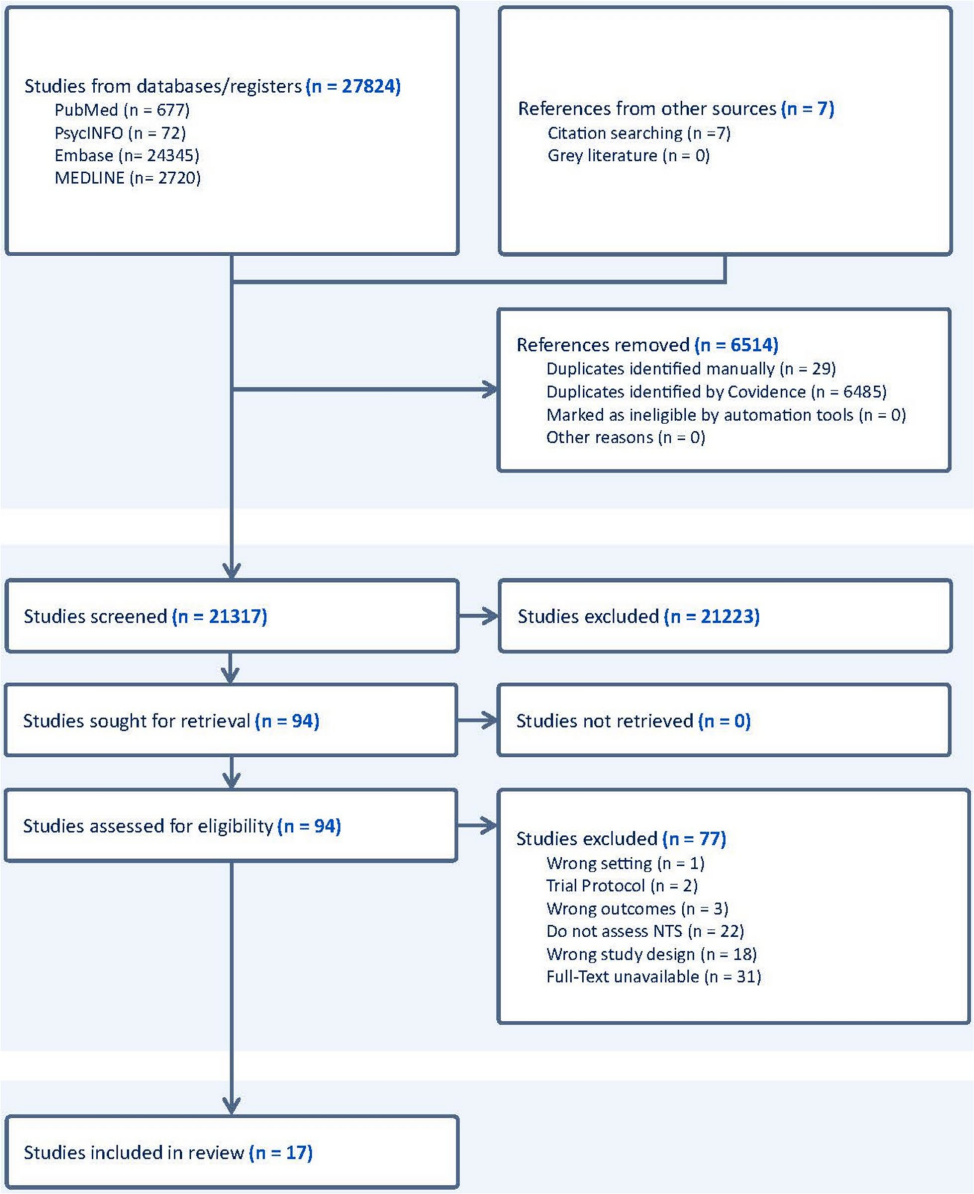
PRISMA flowchart (preferred reporting items for systematic review and meta-analysis)

The search databases yielded 27,824 studies, and seven more were added manually. 17 studies met the inclusion criteria and were fully analysed (PRISMA diagram—Fig. 1).

Twelve were cohort studies; two were cross-sectional studies (surveys), one was a qualitative study (focussed interviews), one was a randomised control trial (RCT), and another involved multiple methods. Eight were performed in a live operating room (OR), seven in a simulated OR and one was performed in a simulation lab using a dry model. Seven of the eight studies performed in the live OR were based on urological procedures, one was performed on gynaecological procedures and in two studies observations included general and colorectal surgeries. In four studies, participants were all experts, two involved only novices, six involved participants with different levels of experience (novice, intermediate and expert), and the experience level was unspecified in five studies (Table 3).

**Table 3:** Summary of Included Studies on Non Technical Skills in Robotic-Assisted Surgery

Study ID	Study design	Number of participants	Participant experience	Study setting	Level of evidence (ocebm)	Name of assessment tool	Type of assessment tool	Nts domain/construct tested	Evaluator type	Mmersqi score
Klein 2009 [16]	Non-randomised experimental	15	Novice	Simulation Lab (Dry)	2b	- Interval Production Task - MRQ - NASA-TLX	Subjective	Cognitive load- Situational awareness	Self-rated	48
Myklebust 2020 [35]	Qualitative research	9	Novice; Intermediate Expert	N/A	3	Semi-structured interview	Subjective	1. Experience of participation in teamwork 2. Positive and negative experiences with teamwork 3. Practical challenges experienced 4. Contributions that can be made to improve teamwork	Expert	N/A
Zattoni 2015 [26]	Non-randomised experimental	7	Expert	OR (Simulation)	3	N/A	Objective	Conversion time and number of errors Improving leadership, clearly defining roles, improving knowledge base and surgical room organisation reduced the occurrence of errors during open conversion	N/A	N/A
Ahmed 2019 [17]	Cross sectional	45	Novice; Intermediate Expert	OR (Intra-operative)	3	- NOTTS - Team performance - Inconveniences - NASA-TLX - Clinical parameters	Objective	Situational awareness, Decision Making, Communication and Teamwork, Leadership and Anticipation	Self-rated Expert	75
Schiff 2016 [24]	Cross sectional	32	Novice; Intermediate Expert	OR (Intra-operative)	3	Quality of Communication Questionnaire	Subjective	Individual Communication Skills, Teamwork, Efficiency, and Provider Satisfaction	Self-rated	60
Raison 2017 [28]	Cohort Study	73	Novice; Intermediate Expert	OR (Simulation)	4	ICARS	Objective	Situational Awareness, Decision Making, Task Management, Leadership, Communication and Team Work, WHO Checklist Completion, Console Set Up and Stress and Distractors	Expert	78
Tiferes 2016 [23]	Other	37	Expert	OR (Intra-operative)	3	Comprehensive analysis	Subjective Objective	Team communications, surgical flow, procedural interruptions	Novice	63
Raison 2018 [36]	RCT	64	Novice	OR (Simulation)	1b	- NOTTS - GEARS - MIQ	Objective	Situational Awareness, Communication and Teamwork, Decision Making and Leadership	Expert	82
Onofrio 2020 [25]	Multivariate analysis	Nil	Expert	OR (Simulation)	3	Modified HEART	Subjective	Communication, Teamwork, Leadership, Decision Making and Situational Awareness	Expert	61
Al jamal 2021 [19]	Cohort	6	Not specified	OR (Simulation)	2b	- ICARS - NASA-TLX	Subjective Objective	Communication and Team Skills, Leadership, Decision-making, Situational Awareness, Stress and Distractors	Expert	65
Melnyk 2022 [28]	Cohort	15	Intermediate Expert	OR (Simulation)	4	Emergency undocking curriculum	Subjective Objective	Communication and Team Working, Decision Making, Leadership and Situational Awareness	Self-rated	70
Norasi 2022 [20]	Non-randomised experimental	4	Expert	OR (Intra-operative)	2b	- NASA-TLX - Modified NOTECHS	Subjective	1. NASA-TLX Task load (mental, physical and temporal demand, performance, effort, frustration, surgeon’s fatigue) 2. Teamwork modified NOTECHS Surgeon’s Leadership and Management, Team’s Teamwork and cooperation, Communication, Problem-Solving and Decision Making, Situational Awareness and Overall Quality of Each Surgery	Self-rated N/A	62
Schreyer 2021 [29]	Multi method observational	Not specified	Not specified	OR (Intra-operative)	2b	RAS-NOTECHS	Objective	Leadership and Management, Teamwork and Cooperation, Problem-solving and Decision making and Situational Awareness	Expert	78
Cha 2022 [31]	Cohort	43	Not specified	OR (Intra-operative)	2b	Sensor based behaviour metrics	Objective	1. Cognitive- Situational Awareness and Decision Making 2. Social- Leadership, Teamwork and Communication 3. Personal Resource- Workload Distribution and Stress Management	Expert	75
Zattoni 2017 [33]	Non-randomised experimental	20	Not specified	OR (Simulation)	2b	Structured training program	Objective	Communication and Teamworking, Leadership and Situational Awareness	N/A	69.5
Manuguerra 2021 [30]	Cohort	N/A	Intermediate Expert	OR (Intra-operative)	2b	NTSRS	Subjective Objective	1. Environmental Dimension- Organisation, ergonomics and disruptors 2. Skills Dimension- Safe communication, Situational Awareness, Cooperation, Leadership and Decision-making	Self-rated Expert	81
Sexton 2018 [18]	Cohort	27	Not specified	OR (Intra-operative)	3	- Recordings (video/audio) - NASA-TLX - Questionnaire	Subjective	Anticipation, teamworking, communication and cognitive load	Self-rated Novice Expert	72

*OR* operation room, *RCT* randomised control trial, *OCEBM* Oxford centre for evidence-based medicine, *MRQ* multiple resources questionnaire; *NASA-TLX* NASA-Task Load Index, *NOTSS* non-technical skills for surgeons; *GEARS* Global Evaluative Assessment for Robotic Skills, *ICARS* Interpersonal and Cognitive Assessment for Robotic Surgery behavioural rating system, *MIQ* Motor Imagery Questionnaire, *HEART* human error assessment and reduction technique, *NOTECHS* non- technical skills, *RAS-NOTECHS* robotic-assisted surgery-non-technical skills, *NTSRS* non-technical skills in robotic surgery

## Domains of non-technical skills

### Cognitive load

Four studies reported on the use of NASA-TLX (National Aeronautics and Space Administration-Task Load Index) to assess cognitive workload across multiple professions [16–20]. NASA-TLX is a subjective (self-administered) questionnaire with six mental dimensions, mental demand, physical demand, temporal demand, effort, performance and frustration [21]. MRQ (Multiple Resources Questionnaire) is another self-administered questionnaire assessing cognitive workload in 17 dimensions and mainly tests auditory, visual, spatial, facial, and tactile resources and short-term memory (STM). While NASA-TLX is sensitive for measuring mental workload in single-task and dual-task parameters, MRQ was able to predict performance breakdown during multitasking situations such as RAS [22].

Klein et al., assessed mental workload in a simulated task using MRQ and NASA-TLX questionnaires and found specific cognitive resources such as recognising visual words, letters, or multiple digits, matching visual letters by rhymed endings and recognising auditory words, digits, or syllables processing were found to be more available compared to other cognitive resources [16].

### Teamwork and communication

Tiferes et al. analysed audio and video recording of team members during live RAS and showed that the most common mode of communication between the surgeon and the two bedside assistants was non-verbal, helped by the console’s shared view on four flat screens [23]. Most movements happened in the zone surrounding the circulating and scrub nurse (*n* = 42, 29%). Interruptions constituted 20% of the total operative time; though they were unlikely to cause adverse outcomes by themselves, the accumulation of minor disruptions had a cumulative effect on increasing susceptibility to surgical error [23]. High levels of ambient noise, problems with the console microphone, console-to-bedside communication and lack of familiarity among team members decreased the quality of communication. Schiff et al., identified that for every one standard deviation (SD) increase in the deficit in the quality of communication perceived, there was an additional 51 ml of estimated blood loss and a 31-min increase in operative time [24]. The modified human error assessment and reduction technique (HEART), was evaluated by Onofrio and Trucco incorporating uncertainties related to personal, team and organisational factors on the surgeon’s human error probability and found that team-related factors such as noise and ambient talk had the highest impact on a surgeon’s performance during RAS and led to increasing the risk of complications during surgery [25].

Video analysis of 20 RAS procedures performed by independent raters, assessed the team’s performance using the Non-Technical Skills for Surgeons (NOTSS) tool and analysed anticipation (tasks performed by the theatre team without or before any verbal request from the surgeon) and inconveniences (tasks involving communication breakdowns or repetitions). This demonstrated that the team’s anticipation had a strong direct correlation with surgeons’ situational awareness and an inverse correlation with the surgeon’s decision-making, communication and teamwork scores. The team’s inconveniences was strongly correlated with higher decision-making and poor leadership and situational awareness scores of the surgeons. A positive association was observed between the NOTSS scores of the surgeon and the team’s self-reported mental and physical demand, physical effort and performance. One study concluded that improving surgeons’ NTS and increasing team familiarity through experience could increase team anticipation and reduce inconveniences during RAS [17]. Additionally, Sexton et al. investigated the importance of anticipation of surgical workflow in RAS. Higher anticipation ratio, was defined as “the ratio of anticipated requests to the total number of requests during a given operation” and showed a significant correlation with shorter operative time (*r* = − 0.44, *p* = 0.01) [17]. In contrast, more requests were associated with longer operative time (*r* = 0.79, *p* < 0.001) [18].

### Team training for emergency situations in RAS

Zattoni et al. reported that 20 simulations of emergency open conversion and reorganisation of the operating theatre during robotic-assisted radical prostatectomy (RARP) reduced time to conversion by 55.2%, increased leadership and improved role delineation. A significant correlation was observed between time to conversion and the number of errors (*R*^2^ = 0.6669). 70% of errors were due to a lack of task sequence, and 50% were due to spatial conflict and loss of sterility. While communication errors, lack of leadership and accidental falls of surgical devices were the others reported events. Four strategies were implemented following the first simulation to improve the workflow. These include improving leadership, clearly defining roles, improving the knowledge base, and reorganising the operating theatre [26]. Melnyk et al. developed a curriculum where participants were exposed to a full-immersion simulation that activated the Emergency Robotic Undocking Protocol. Various surgical metrics were used including time to undock and surgeons’ electrodermal activity were calculated. After the program was finished, surgeons’ knowledge and confidence in emergency undocking grew significantly, substantially decreasing mistakes during simulations and, therefore, higher action scores and shortened decision-making time [27]. Al Jamal et al. implemented and evaluated a simulation-based Robotic Colorectal Surgery Non-Technical Skills Robotic training and assessment curriculum consisting of two scenarios: pelvic bleeding and CO2 embolism, tested in a simulated operating theatre. At the start of the first session, the resident’s self-rated ICARS scores were lower than the expert-rated scores. Nevertheless, six months later, at the second session, both self-rated and expert scores coincided in all NTS categories [19].

## NTS assessment tools specific for RAS

Three novel rater-based objective assessment tools for assessing NTS in RAS were identified, namely: (i) ICARS (Interpersonal and Cognitive Assessment for Robotic Surgery) behavioural rating system; (ii) RAS-NOTECHS (Robotic-Assisted Surgery-Non-Technical Skills), and (iii) NTSRS (Non-technical Skills in Robotic Surgery) [28–30] (Table 4). The ICARS and NTSRS, for console and bedside surgeons and RAS-NOTECHS, of the entire RAS team.

**Table 4:** Messick’s validity of novel rater-based assessment tools for NTS (non-technical skills) in RAS (robotic-assisted surgery)

Name of assessment tool	Concurrent validity	Internal consistency	Inter-rater reliability	Construct validity	Difference between novice and expert	Factor analysis
1. ICARS (Interpersonal and Cognitive Assessment for Robotic Surgery behavioural rating system)	• High degree of correlation between ICARS and NOTSS (gold standard) • Bland–Altman analysis showed narrow 95% confidence interval (Z score -0.66 to 0.65) with uniform scatter of plots indicating good agreement	• The checklist demonstrated a reliable internal structure • All the five categories demonstrated high alpha coefficients (median = 0.92; range 0.85–0.94)	• Direct comparison performed using interclass coefficient (ICC) showed greater degree of agreement between raters with a mean ICC of 0.60	• ICARS Scores can reliably assess various NTS constructs identified during the development phase	• ICARS Scores reliably differentiate experts from novice participants • However, disproportionately low number of experts compared to novices used during tool development	• High level of acceptance (80%) amongst the panel of experts that ICARS actively assessed NTS and identify deficits using • The ICARS score is able consistently rates key NTS behaviours during RAS
2. RAS-NOTECHS (Robotic-Assisted Surgery-Non-Technical Skills)	• Scores lacks concurrent validity as no comparison was made with scores of established tools like NOTECHS II	• Moderate degree of Inter class correlation (ICC) was found for scores of the complete RAS-NOTECHS (ICC 0.687, 95% CI [0.639; 0.729])	• The study reported strong inter-rater agreement for the list of individual behaviours • Gwet’s AC1 of 0.831, 95% CI [0.789, 0.874]	• The tool was able to reliably test and measure RAS-specific teamwork behaviour	• Not Available	• Not Available
3. NTSRS (Non-technical Skills in Robotic Surgery)	• Scores had a very strong negative correlation with number of near misses (r = − 0.92, *p* < 0.001) • Experience of the surgeon had a moderate correlation with decision making, i.e. number of RAS procedures (r = 0.45, *p* < 0.05) and years of experience in RAS (r = 0.4, *p* < 0.05)	• Environmental and skill dimensions showed statistically significant correlation (correlation coefficient, r = 0.59, *p* = 0.002) • ICC was excellent for both grades of near-miss events. For Grade 1 it was 0.87, 95% CI [0.75; 0.94] and 0.80, 95% CI [0.61; 0.90] for Grade 2	• Inter-rater reliability was not measured	• The tools show good internal consistency • Poor NTSRS scores correlated with an increase in near-miss events • Surgeon’s experience was not correlated to NTSRS scores except in the area of decision making	• No correlation was analysed between experts scores and self-rated scores	• Surgeon’s experience needs to be distinguished from proficiency in overall NTS

The ICARS (Interpersonal and Cognitive Assessment for Robotic Surgery behavioural rating system) tool was developed in the UK, and it incorporated four key NTS domains and seven categories [28]. New domains such as WHO Checklist Completion, Console Setup and Distractors were included along with generic domains of situational awareness, decision-making, task management, leadership and communication and teamwork. An observational trial involving participants with novice, intermediate or expert proficiency in RAS was performed in a high-fidelity simulated operating room environment. A panel of experts then assessed the videos of all participants using the ICARS and NOTSS [28]. The validity and reliability of ICARS Scores were proven to assess various NTS constructs identified (Table 4). The drawback of this tool is that it explicitly measures the NTS of the console surgeon and not the rest of the RAS team.

The RAS-NOTECHS (Robotic-Assisted Surgery-Non-Technical Skills) tool was developed in Germany by Schreyer et al., based on the Oxford NOTECHS II (Oxford Non-Technical Skills), with a strong emphasis on creating an NTS assessment for the multi-professional RAS team. Using this tool, two trained, independent observers rated the NTS of the operating team in six urological RAS procedures simultaneously [29]. OR teamwork behaviour is an essential factor in the safety and quality of surgical care. RAS-NOTECHS can be applied while observing real-life procedures and correlated with operative and patient outcomes.

The NTSRS tool (Non-technical Skills in Robotic Surgery) was developed by Manuguerra et al., in France as part of a multi-centre study to assess the NTS of surgical teams in RAS and their relationship with the occurrence of near-miss events in a live-operating theatre setting [30]. In addition to existing NTS, this tool includes an ‘environment’ domain which is further divided into organisation, ergonomics of the operating room, and stress and disruptors management. High scores were associated with a significant reduction in near-miss events. The authors noted that the surgeon’s experience did not correlate with NTSRS scores except in decision-making, highlighting the importance of training in NTS [30].

One study identified a more objective method using sensor-based measurement of surgeons’ communication, speech and proximity metrics demonstrating a good correlation with NOTSS scores [31]. Focusing only on the NTS of the console and bed-side surgeons, it had a more objective approach and demonstrated its more cost-effectiveness [31].

## Discussion

Our findings show that maintaining a dedicated team, addressing environmental factors, and routine team training by simulating intraoperative emergencies is important for safe RAS procedures. We also found one sensor-based, two subjective and three rater-based NTS assessment tools for RAS. Further research is required to assess the validity and improve the generalisability of these assessments.

Surgeons and assistants performing RAS procedures experience high cognitive strain, especially during the early stages of their learning curve when working with a new platform or an unfamiliar team. Team-related factors such as ambient noise and chatter, inconveniences due to repeated requests during the procedure and constraints due to poor design of the OR may have an adverse effect on patient safety during RAS. This systematic review has identified studies demonstrating that team familiarity and training can improve communication and increase anticipation during RAS. Addressing environmental factors by reducing ambient noise and disruptions will improve workflow and readiness during emergencies.

Anticipation represents the highest level of a shared mental model where the team can predict and execute the next move before an explicit verbal command by the surgeon. So, there is less verbal communication and a need for decision-making by the surgeon [18]. Environmental factors such as high ambient noise levels, obstructions to direct line of sight by robotic components, and lack of space are unique to RAS. Utilising these discoveries, OR team’s working conditions can be improved to reduce the impact of disruptions and avoid performance drops caused by multitasking in a RAS environment. Ideally, it would be better to have purpose built RAS-specific operating rooms; however, this might not always be feasible.

Routine adoption of the methodology provided by Tiferes et al. when installing surgical robots in a “traditional” OR will help identify congestion points, improve flow and resolve disruptors, as this has important implications during intraoperative emergencies [23]. Recognising critical steps during surgery and making it evident to the entire surgical team will reduce chatter or disruptions from movement in and out of the OR and help the team develop a shared mental model. Secondly, identifying different error modes and their recovery actions, as suggested by Onofrio and Trucco, would help with training and improvement of patient safety [25]. Another exciting possibility is using artificial intelligence and machine learning technology to predict potential errors in advance or prompt recovery actions when errors occur.

NTS has a particular emphasis in RAS procedures, given the increased cognitive workload on the surgeon and the team. According to the 2015 European Association of Endoscopic Surgeons (EAES) consensus statement on using robotics in general surgery, “Robotic systems need a dedicated team with special training”. Hence team familiarity is an important factor which should be considered when scheduling staff [32]. Nonetheless, in hospitals and healthcare systems where it cannot be practical to keep a devoted team, it is essential to cultivate a core team of able persons.

Implementing robotic NTS training during intraoperative emergencies was examined by four studies, looking into designing and implementing a curriculum for surgeon and team training during intraoperative emergencies requiring conversion to open surgery [19, 26, 27, 33]. Team training on crisis-resource management should be performed regularly by RAS teams even though these are rare events. For the successful adaptation of newer RAS systems, research must be expanded to address issues relating to the interaction between the surgeon, surgical team, and the new technology. Seemingly, surgeons’ technical competence has been assigned higher importance due to the direct implications of a performed error and its adverse outcomes. However, near-miss events increase when working under stress, which can have a cumulative effect on patient safety.

This systematic review highlighted the paucity of reporting on NTS in robotic surgery, identifying only three bespoke objective assessment tools. These three tools were utilised in four studies, while the remaining papers reported on either subjective or sensors-based tools that are not specific for robotic-assisted surgery. These objective tools, nevertheless, have shown that important traits such as situational awareness, decision-making, task management, leadership and communication and teamwork can be objectively measured, which can help to identify gaps and offer remedial training to individuals and teams. The tools can also help educators and researchers develop sound training curriculums and improve patient safety.

All evidence supports that ICARS Scores can reliably assess various NTS constructs identified during its development phase. The drawback of this tool is that it explicitly measures the NTS of the console surgeon and not the rest of the RAS team [28]. Similar to ICARS, the NTSRS tool includes an ‘environment’ domain, which is further divided into the organisation, ergonomics of the operating room, and stress and disruptors management. High scores were associated with a significant reduction in near-miss events. The authors also note that the surgeon’s experience did not correlate with NTSRS scores except in decision-making; hence, experience must be distinguished from specific training in NTS [30]. The RAS-NOTECHS is a promising tool that could be used to implement training and design curriculum for all team members and potentially can be tested for other robotic systems however, the scores lack concurrent validity as no comparison was made with scores of established tools like NOTECHS II [29].

The ICARS score has the best validity evidence available to reliably assess the NTS of the console surgeon during RAS procedures. There are positive signs that the ICARS tool is becoming a standard for assessing surgeons’ NTS in RAS and being adopted into RAS training curricula; hence, we recommend its use. However, the RAS-NOTECHS is ideal for testing the NTS of the entire operating team, and as emphasised earlier, team-related factors weigh heavily on the surgeon’s cognitive load.

A more objective method using sensor-based measurement of surgeons’ communication, speech and proximity metrics showed a good correlation with NOTSS scores [31]. Again, the study focused on measuring the NTS of the console surgeon and the bedside surgeon. However, the method described in the study is less resource-heavy and more objective than traditional rater-based systems. Sensor-based measurements provide a foundation for further research and development to evaluate the NTS of the entire RAS team with additional physiological parameters in real time.

The most commonly used subjective assessment tool in this review was NASA-TLX, which has been used in multiple studies to assess cognitive load of the surgeon as well as team members [16–20]. One study demonstrated that a surgeon and operating team with with considerable RAS experience, exhibited significant cognitive strain when performing the same procedure using a different type of RAS platform [20]. Cognitive strain remained high even after completing twenty similar procedures with the new platform. Hence, careful consideration for research and planning is essential prior to and during the introduction of new technology or robotic platforms, as there is a risk of introducing human error that could affect a surgeon or OR teams learning curve, potentially risking patient safety. In addition, it may affect decisions on what the minimum number of procedures is by the RAS team to overcome its learning curve [20]. Additional support in the form of remote proctoring, technical expertise and minimising external stressors is essential to reduce the incidence of near misses or adverse events during the learning curve. Also, the surgeon’s experience does not correlate with NTS; hence it would be wrong to assume that more experienced operators have good NTS. When operators experienced higher cognitive load, their vocal, auditory and visual resources were more available than others [16]. Hence, reducing ambient noise or disruptions from movement in and out of the OR is essential.

While NTS are general to all surgical specialities, the current evidence is limited predominantly to robotic urological surgeries. However, the situational awareness, speed of the surgeon, duration of procedure, and complexity vary in different surgical procedures.. For example, operative events that may occur during prostatectomy can be different from those which may occur during robotic colorectal surgery. Additionally, the context of operating on one limited zone/ region can be different from operating on multivesceral surgery and the challenges that can be associated with each type of surgery can be different which may influence the NTS. Rather than developing new tools, adopting and further evaluating existing ones is essential. Evaluation will need to extend to new and emerging RAS platforms, since all the studies in this review used the Da Vinci robotic system. Other robotic platforms differ in design and ergonomics; for example, some newer RAS systems have an open console and occupy less space than existing systems [3], therefore, representing potential novel challenges or resolutions to effective NTS. RAS is a highly complex environment; hence it is essential to evaluate the NTS of the entire multi-professional team comprehensively [29].

**Fig. 2:**
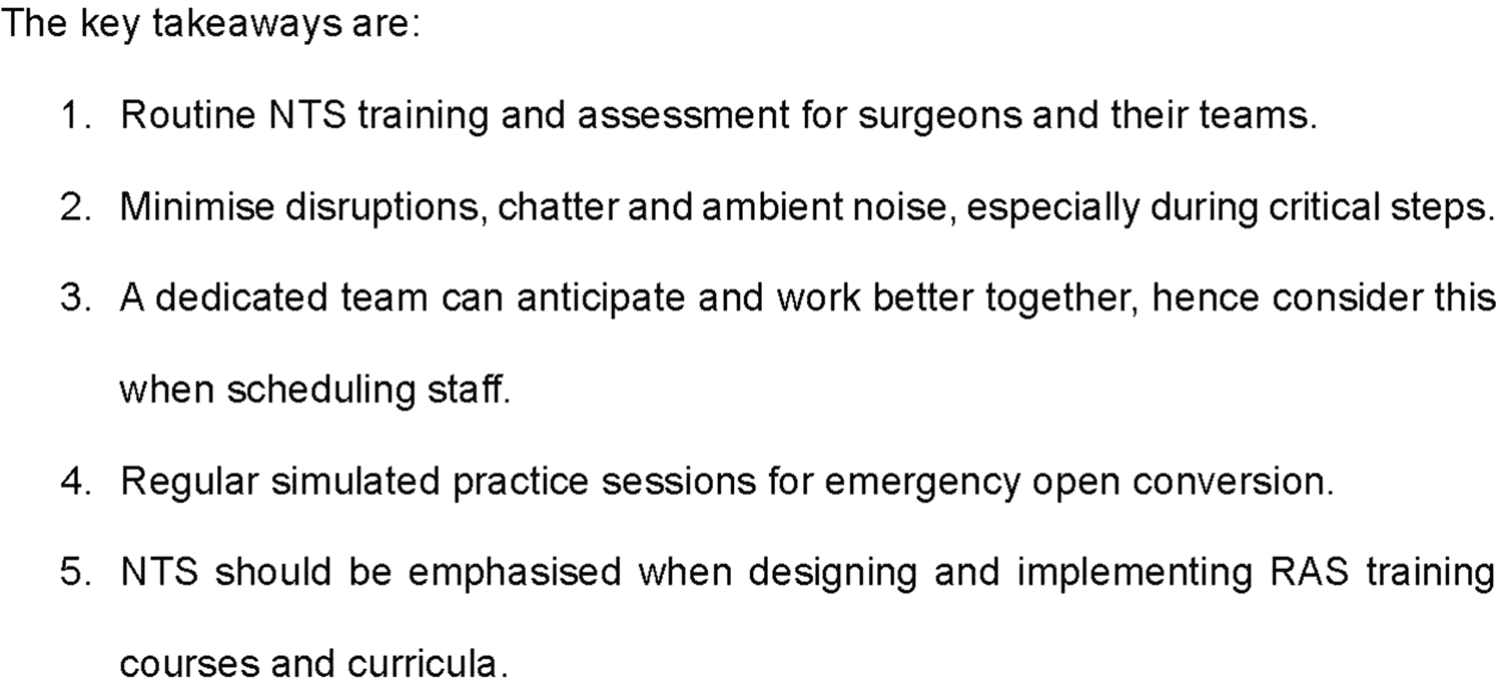
Key takeaways

Limitations of this systematic review include the possibility of missing some unpublished literature and 31 articles were removed as the full text was unavailable. A search of the “grey” literature was not performed due to the large number of titles for screening and time constraints. As a result, publication bias could not be convincingly excluded. Further examination into the implementation of sensor-based measurements utilising distinct physiological parameters with increasingly miniaturised measurement devices will provide a more objective and immediate assessment of NTS. Progression in the design of systems and instruments will facilitate communication between the surgeon and the rest of the team, promoting the formation of a shared mental model during the procedure. The development of telesurgery necessitates the formation of a global, high-fidelity, emergency robotic undocking curriculum, akin to the ATLS (Advanced Trauma Life Support) [34]. Investigating the most advantageous theatre design and set-up, which can diminish crowding and enhance productivity (Refer Fig. 2—Key takeaway).

## Conclusion

This systematic review has highlighted multiple non-technical skills tools, with three main ones, most of which are under evaluated. Whilst promising, increased awareness and widespread use across multiple specialities is lacking. Further evaluative research is required to report on incorporating non-technical skills training and assessment in robotic surgery curricula, to demonstrate the potential benefits and improve patient safety in robotic surgery.
